# A novel human coronavirus: Middle East respiratory syndrome human coronavirus

**DOI:** 10.1007/s11427-013-4519-8

**Published:** 2013-08-07

**Authors:** HeYuan Geng, WenJie Tan

**Affiliations:** Biotech Center for Viral Disease Emergency, Key Laboratory of Medical Virology and Viral Diseases Prevention and Control, Ministry of Health, National Institute for Viral Disease Control and Prevention, Chinese Center for Disease Control and Prevention, Beijing, 102206 China

**Keywords:** HCoV-EMC, MERS-CoV, genomic characterisation, molecular detection

## Abstract

In 2012, a novel coronavirus, initially named as human coronavirus EMC (HCoV-EMC) but recently renamed as Middle East respiratory syndrome human coronavirus (MERS-CoV), was identified in patients who suffered severe acute respiratory infection and subsequent renal failure that resulted in death. Ongoing epidemiological investigations together with retrospective studies have found 61 laboratory-confirmed cases of infection with this novel coronavirus, including 34 deaths to date. This novel coronavirus is culturable and two complete genome sequences are now available. Furthermore, molecular detection and indirect immunofluorescence assay have been developed. The present paper summarises the limited recent advances of this novel human coronavirus, including its discovery, genomic characterisation and detection.

Coronaviruses, a genus in the family Coronaviridae (order Nidovirales), are a large family of viruses that can cause respiratory, enteric, hepatic, nephric and neurological diseases with variable severity in humans and animals. In humans, diseases caused by coronaviruses range from the common cold to severe acute respiratory syndrome (SARS). Based on genome sequence analyses, coronaviruses are phylogenetically divided into three genera known as *Alph*-, *Beta*- and *Gammacoronavirus*. *Betacoronavirus* contains four subgroups, named 2a, 2b, 2c and 2d. A fourth genera, provisionally named *Deltacoronavirus*, comprises a number of recently identified coronaviruses mainly from birds. *Alph*- and *Betacoronavirus* hosts are predominantly domestic mammalian animals and humans, including swine (transmissible gastroenteritis virus, TGEV; porcine epidemic diarrhoea virus, PEDV), bovine (bovine coronavirus, BCoV), mice (mouse hepatitis virus, MHV), dog (canine coronavirus, CCoV), cat (feline infectious peritonitis virus, FIPV) and bat (bat coronavirus HKU4, BatCoV-HKU4). Human coronaviruses (HCoVs) known so far include HCoV-229E, HCoV-OC43, HCoV-HKU1, HCoV-NL63 and SARS (SARS-CoV). *Gammacoronavirus* comprises primarily avian infectious bronchitis virus (IBV) and beluga whale coronavirus SW1 (BWCoV-SW1) [1]. The emerging of new coronaviruses has been expanding the taxonomic diversity of coronavirus.

There are several factors resulting in new coronaviruses or subtypes continual emerging. These factors include the infidelity of RNA-dependent RNA polymerase (RdRp), a high gene mutation and recombination rates, the occurrence of site-specific or random mutations via a template switching mechanism during RNA replication and transcription, together with a large viral genome and a wide range of hosts.

In addition, these factors facilitate adaptation to new hosts and ecological niches that may cause zoonotic diseases events.

Several new coronaviruses have been identified in the past decade, e.g., SARS-CoV outbroke during 2002–2003 [2]; HCoV-NL63 and HCoV-HKU1 isolated in 2004 [3] and in 2005 [4] respectively; the SARS-like-CoVs isolated from bats [5]; the alpaca coronavirus (ACoV) isolated in 2007 [6] and the novel rabbit coronavirus HKU14 isolated from domestic rabbits in 2012 [7]. It is worth noting that in June 2012, a novel human coronavirus, Middle East respiratory syndrome human coronavirus (MERS-CoV), was isolated in the Middle East and to date 34 of the 61 laboratory-confirmed cases of MERS-CoV infection have died as of June 14th (www.who.org) [8,9]. The present paper summarises recent advances in MERS-CoV isolation, genome characterisation and molecular detection.

## 1 Identification of MERS-CoV

### 1.1 Case 1

In June 2012, a Saudi man in his sixties got an acute respiratory tract infection in Saudi Arabia with symptoms of fever, cough, expectoration and shortness of breath. The patient who was previously well, was severely ill and presented with acute pneumonia and later renal failure with a fatal outcome 11 days after hospitalization [10]. Clinical specimens were collected and the patient's sputum was used to inoculate Vero and LLC-MK2 cells for viral culture. Cytopathic effect (CPE) was observed and, upon passaging of the culture supernatant to fresh cells, the same CPE was observed, suggesting viral replication. All known respiratory tract pathogens including the influenza A virus, influenza B virus, adenovirus, enterovirus, parainfluenza viruses types 1–3, respiratory syncytial virus, human metapneumovirus, human bocavirus, rhinovirus, and human herpesvirus types 1–3 were tested and all yielded negative results from the sputum and cell culture supernatant. However, the pan-coronavirus real-time reverse transcription polymerase chain reaction (RT-PCR) assay used to detect coronaviruses revealed the expected size of the PCR fragments, and found that they corresponded to a conserved region of ORF1b of the replicase gene of a coronavirus. Phylogenetic analysis of this fragment with other known coronaviruses genes showed that this novel virus together with the bat coronaviruses HKU4 and HKU5 belonged to subgroup 2c of the linage *Betacoronavirus*, indicating the emergence of a novel coronavirus [11]. The complete genome of the virus was subsequently sequenced at the Erasmus Medical Cerner (EMC) in Rotterdam in the Netherlands and was named human coronavirus EMC (HCoV-EMC), later named MERS-CoV (GenBank accession number: JX869059).

### 1.2 Case 2

On 11 September 2012, a Qatari man in his forties with severe respiratory illness was transferred from Qatar to a United Kingdom hospital and was admitted to the intensive care unit for progressive hypoxia and renal failure. Extensive diagnostic tests on upper and lower respiratory tract samples for a causative agent were negative. The pan-coronavirus RT-PCR assay, however, yielded a correct band size (208 bp) that corresponded to the conserved RNA dependent RNA polymerase gene of coronavirus in lower respiratory tract specimens. Assays for detection of known human coronaviruses HCoV-OC43, -229E, -NL63, -HKU1 and SARS-CoV were negative [12]. A comparison of the PCR fragment sequence with the nucleotide sequence of HCoV-EMC isolated from case 1, showed that they shared 99% identity, indicating the same viral infection. Subsequently, the complete genome sequence of this virus was sequenced in London and called *Betacoronavirus* England 1 (GenBank accession number: KC164505) [13].

### 1.3 More Cases

In retrospective studies, two cases of infection with MERS-CoV in Jordan were confirmed. These cases were discovered by testing stored specimens from a cluster of pneumonia cases in healthcare workers that occurred in April 2012. Both of the confirmed cases have died. To date, a total of 46 laboratory confirmed cases have been reported in Saudi Arabia (including 28 deaths). Jordan, Qatar, the United Arab Emirates (UAE), France, Germany, Italy, Tunisia and the United Kingdom also reported laboratory-confirmed cases. The common features of confirmed MERS-CoV infection are fever, cough, shortness of breath presented with acute pneumonia. Acute renal failure occurred in fatal cases.

## 2 Genome characterisation and phylogenetic analysis

Coronaviruses are the largest known enveloped positive-stranded RNA viruses, ranging in length from 26 to 32 kb. A terminal cap at the 5′ end is followed by a leader sequence of 65–98 nucleotides (nt), and an untranslated region (UTR) of 200–600 nt. The leader sequence is present at the 5′ end of all subgenomic mRNAs. Another UTR of 200–500 nt is at the 3′ end of the genome followed by a poly(A) tail of variable length. 5′-UTR contains the signals for genome replication, transcription and encapsidation, whereas the 3′-UTR contains transcription termination signals [14]. At the 5′ end nearly two-thirds of the genome encode two large overlapping open reading frames (ORF), ORF1a and ORF1b. When the coronavirus enters the host cell, ORF1a and ORF1b are first translated into two large polyproteins, pp1a and pp1ab. Production of pp1ab requires a programmed −1 ribosomal frameshift between the ORF1a and ORF1b junction. These polyproteins are processed autoproteolytically and subsequently form a transcription-replication complex [15]. About one-third of the genome at the 3′ end encodes four structural proteins, named the spike protein (S), envelop protein (E), small membrane protein (M) and the nucleocapsid protein (N). These structural proteins are present in all known coronaviruses. The general genome organisation of coronaviruses is as follows: 5′-UTR-1a/1b-spike (S)-envelope (E)-membrane (M)-nucleocapsid (N)-3′-UTR. Among the structural proteins are several small group-specific ORFs, which differ markedly in number and gene order, but are conserved within the same group. These small ORFs are dispensable for viral replication *in vitro*, but determine viral pathogenesis [16].

The novel coronavirus MERS-CoV is culturable and two complete genome sequences are now available. The complete genome sequence of the novel MERS-CoV contains 30119 nt and at least 10 predicted ORFs, which is similar to other HCoVs. The 5′-UTR and 3′-UTR contain 278 and 300 nt, respectively. Near the 5′ end, about 3/4 of the genome is occupied by two large ORFs, ORF1a (279–13433 nt) and ORF1b (13433–21514 nt). These two ORFs are translated from genomic mRNA to produce polyproteins pp1a and pp1ab by ribosomal frameshifting at the junction of ORF1a and ORF1b, with nt 13427 to 13433 predicted to form the "slippery sequence" (5′- UUUAAAC-3′) to produce polyproteins pp1ab. Downstream of ORF1b, the genome encodes four structure proteins, including the spike (S), envelope (E), membrane (M) and nucleocapsid (N) [17]. Four small non-structural ORFs (3a, 3b, 3c, 3d) lie between the S and E genes. The gene order of MERS-CoV is as follows: 5′-UTR-1a/1b-S-ORF3 (formed to 3a, 3b, 3c, 3d)-E-M-N-3′-UTR, which most closely resembles the gene structure of BatCoV-HKU4 and BatCoV-HKU5 (Figures 1 and 2).

### 3 Laboratory detection of MERS-CoV

To date, at least three real-time (RT-PCR) assays to screen and confirm MERS-CoV infection have been developed by Europe laboratories. The first RT-PCR screening assay (upE assay) was developed to target regions upstream of the E gene (27458–27550 nt), with sensitivity of 3.4 copies per reaction. Subsequently, a confirmatory assay targeting the ORF1b gene was developed, which does not overlap with the pan-coronavirus RT-PCR assays [18]. A third, confirmatory real-time RT-PCR assay, developed in Europe laboratories, targeted the ORF1a (1a assay) and its sensitivity was slightly lower than that of the upE assay. Furthermore, an immunofluorescence assay (IFA) to detect the antibody response was developed using convalescent patient serum that was biologically safe [19]. The upE assay used in combination with the 1a assay has been reported to provide a rigorously validated and highly sensitive result [20]. We have designed several primers and probes for real-time RT-PCR, modified using locked nucleic acid (LNA), to target the ORF1b gene with validated high sensitivity and no cross-reaction with HCoV-229E, -OC43, -NL63, -HKU1 and other respiratory pathogens [21]. The primers and probes designed in our laboratory and Europe laboratories are shown in Table 1.

### 4 Summary

To date, a total of 61 laboratory-confirmed cases of infection with MERS-CoV have been reported from Jordan, Qatar, Saudi Arabi, the United Arab Emirates (UAE), France, Germany, Italy and the United Kindom, including 34 deaths. The clinical presentation of acute pneumonia and renal failure observed in the fatal cases resembled SARS-CoV that outbroke in 2002–2003. MERS-CoV does not use the angiotensin converting enzyme 2 (ACE2) as a receptor for cell entry but maintains broad replicative capability in mammalian cell lines from bats, pigs and humans, indicating that MERS-CoV is different from SARS-CoV [22]. Phylogenetically, however, HCoV-EMC is most closely related to the bat coronaviruses HKU4 and HKU5 in the same *Betacoronavirus* genus as SARS-CoV, indicating a zoonotic origin

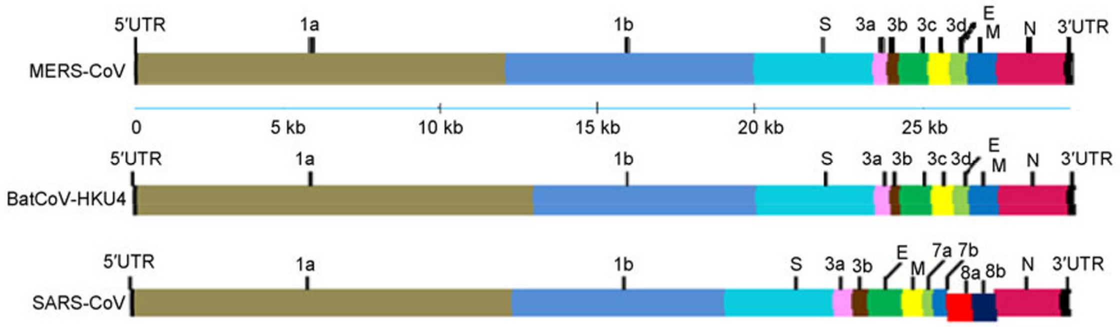

**Figure 1** Genome structure of MERS-CoV, BatCoV-HKU4 and SARS-CoV. Phylogenetic analysis based on the full-length genome sequence of MERS-CoV and other known coronaviruses with available complete genome sequences show that MERS-CoV is in subgroup 2c of the genus *Betacoronavirus*, close to BatCoV-HKU4 and BatCoV-HKU5 (Figure 2).

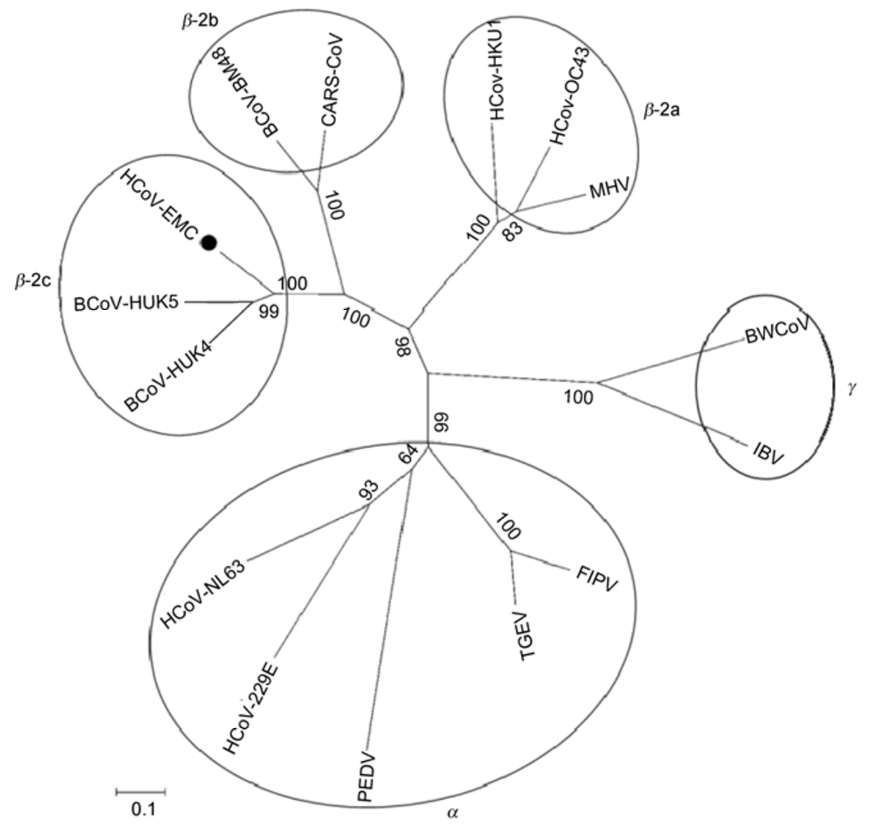

**Figure 2** Phylogenetic tree analysis of MERS-CoV (original name HCov-EMC) based on full-length genome sequence. Alignments were performed using ClustalW and the tree was constructed using the neighbor-joining method with bootstrap value (1000 replications). TGEV, porcine transmissible gastroenteritis virus; PEDV, porcine epidemic diarrhoea virus; IBV, avian infectious bronchitis virus; BWCoV, beluga whale coronavirus sw1; MHV, mouse hepatitis virus; FIPV, feline infectious peritonitis virus; BCoV-HKU4, bat coronavirus HKU4; BCoV-HKU5, bat coronavirus HKU5.

**Table 1** Primers and probes used for screening MERS-CoV infection using real time RT-PCR[a)]

| Primer and probe | Sequence (5′–3′) | Target | Size (bp) |
|---|---|---|---|
| ORF1b-F | ACTGTTGCAGGCGTGTCCATACTTAGC | ORF1b* | 109 |
| ORF1b-R | TAGTACCAATGACGCAAGTCGCTCC | | |
| ORF1b-P** | [FAM]-AGCACAATGACTAATCGCCA-[TAMRA] | | |
| upE-Fwd | GCAACGCGCGATTCAGTT | upE | 92 |
| upE-Rev | GCCTCTACACGGGACCCATA | | |
| upE-Prb | [FAM]-CTCTTCACATAATCGCCCCGAGCTCG-[TAMRA] | | |
| ORF1b-Fwd | TTCGATGTTGAGGGTGCTCA | ORF1b | 82 |
| ORF1b-Rev | TCACACCAGTTGAAAATCCTAATTG | | |
| ORF1b-Prb | [FAM]-CCCGTAATGCATGTGGCACCAATGT-[TAMRA] | | |
| ORF1a-Fwd | CCACTACTCCCATTTCGTCAG | ORF1a | 84 |
| ORF1a-Rev | CAGTATGTGTAGTGCGCATATAAGCA | | |
| ORF1a-Prb | [FAM]-TTGCAAATGGCTTGCCCCCACT-[TAMRA] | | |

a) *, ORF1b: primers and probe designed for real-time RT-PCR in our laboratory; **, ORF1b-P: probe modification using locked nucleic acid (LNA).

[23]. Although experimental detection assays have been developed, no vaccine or antiviral drug for prevention and treatment of MERS-CoV infection is available because only limited cases have been reported thus far. The original source, pathogen biology, transmission and clinical impact of the MERS-CoV infection requires further investigation.

*This work was supported by grants from the National Basic Research Program of China (2011CB504704) and the State Megaproject for Infectious Disease Research of China (2011ZX10004-001).*

1 Virus taxonomy, classification and nomenclature of viruses: ninth report of the International Committee of Taxonomy of Viruses. San Diego, CA: Academic Press, 2012

2 Guan Y, Zheng B J, He Y Q, et al. Isolation and characterization of viruses related to the SARS coronavirus from animals in southern China. Science, 2003, 302: 276–278

3 van der Hoek L, Pyrc K, Jebbink M F, et al. Identification of a new human coronavirus. Nat Med, 2004, 10: 368–373

4 Woo P C Y, Lau S K P, Chu C M, et al. Characterization and complete genome sequence of a novel coronavirus, coronavirus HKU1, from patients with pneumonia. J Virol, 2005, 79: 884–895

5 Lau S K, Woo P C, Li K S, et al. Severe acute respiratory syndrome coronavirus-like virus in Chinese horseshoe bats. Proc Natl Acad Sci USA, 2005, 102: 14040–14045

6 Crossley B M, Barr B C, Magdesian K G, et al. Identification of a novel coronavirus possibly associated with acute respiratory syndrome in alpacas (*Vicugna pacos*) in California, 2007. J Vet Diagn Invest, 2010, 2007, 22: 94–97

7 Lau S K P, Woo P C Y, Yip C C Y, et al. Isolation and characterization of a novel *Betacoronavirus* subgroup A coronavirus, rabbit coronavirus HKU14, from domestic rabbits. J Virol, 2012, 86: 5481–5496

8 Wu Y, Gao G F. Severe fever with thrombocytopenia syndrome virus expands its borders. Emerg Microbes Infect, 2013, in press

9 Zaki A M, van Boheemen S, Bestebroer T M, et al. Isolation of a novel coronavirus from a man with pneumonia in Saudi Arabia. N Engl J Med 2012, 367: 1814–1820

10 ProMED-mail. Novel coronavirus—Saudi Arabia: human isolate Archive Number: 20120920.1302733 20 September 2012 21:51:30 CEST. http://www.promedmail.org/direct.php?id=20120920.1302733

11 Zaki A M, van Boheemen S, Bestebroer T M, et al. Isolation of a novel coronavirus from a man with pneumonia in Saudi Arabia. N Engl J Med, 2012, 367: 1814–1820

12 Bermingham A, Chand M A, Brown C S, et al. Severe respiratory illness caused by a novel coronavirus, in a patient transferred to the United Kingdom from the Middle East, September 2012. Euro Surveill, 2012, 17: 20290

13 Health Protection Agency. HPA Press release. Acute respiratory illness associated with a new virus identified in the UK. London: HPA, 2012. http://www..hpa.org.uk/NewsCentre/NationalPressReleases/2012PressReleases/120923acuterespiratoryillnessidentified/

14 Woo P C, Huang Y, Lau S K, et al. Coronavirus genomics and bioinformatics analysis. Viruses, 2012, 2: 1804–1820

15 Hagemeijer M C, Rottier P J, de Haan C A. Biogenesis and dynamics of the coronavirus replicative structures. Viruses, 2012, 4: 3245-3269

16 McBride R, Fielding B C. The role of severe acute respiratory syndrome (SARS)-coronavirus accessory proteins in virus pathogenesis. Viruses, 2012, 4: 2902–2923

17 van Boheemen S, de Graaf M, Lauber C, et al. Genomic characterization of a newly discovered coronavirus associated with acute respiratory distress syndrome in humans. mBio, 2012, 3: e00473-12

18 Corman V M, Eckerle L, Bleicker T, et al. Detection of a novel human coronavirus by real-time reverse-transcription polymerase chain reaction. Euro Surveil, 2012, 17: 20285

19 Corman V M, Müller M A, Costable U, et al. Assays for laboratory confirmation of novel human coronavirus (hCoV-EMC) infections. Euro Surveill, 2012, 17: 20334

20 Palm D, Pereyaslov D, Vaz J, et al. Laboratory capability for molecular detection and confirmation of novel coronavirus in Europe, November 2012. Euro Surveill, 2012, 17: 20335

21 Zhou W M, Lu R J, Geng H Y, et al. Molecular detection assays for 2012 identified novel human coronavirus (HCoV) and probe modification with locked nucleic acid (LNA) (in Chinese). Zhonghua Shi Yan He Lin Chuang Bing Du Xue Za Zhi, 2012, 26: 401–404

22 Müller M A, Raj V S, Muth D, et al. Human coronavirus EMC does not require the SARS-coronavirus receptor and maintains broad replicative capability in mammalian cell lines. mBio, 2012, 3: e0051512

23 Lu G W, Liu D. SARS-like virus in the Middle East: a truly bat-related coronavirus causing human diseases. Protein Cell, 2012, 3: 803–805

**Open Access** This article is distributed under the terms of the Creative Commons Attribution License which permits any use, distribution, and reproduction in any medium, provided the original author(s) and source are credited.
